# Seneca Valley Virus 3C Protease Inhibits Stress Granule Formation by Disrupting eIF4GI-G3BP1 Interaction

**DOI:** 10.3389/fimmu.2020.577838

**Published:** 2020-09-29

**Authors:** Wei Wen, Qiongqiong Zhao, Mengge Yin, Liuxing Qin, Junjie Hu, Huanchun Chen, Xiangmin Li, Ping Qian

**Affiliations:** ^1^State Key Laboratory of Agricultural Microbiology, College of Veterinary Medicine, Huazhong Agricultural University, Wuhan, China; ^2^Key Laboratory of Preventive Veterinary Medicine in Hubei Province, The Cooperative Innovation Center for Sustainable Pig Production, Wuhan, China; ^3^Hubei Colorectal Cancer Clinical Research Center, Hubei Cancer Hospital, Wuhan, China; ^4^Key Laboratory of Development of Veterinary Diagnostic Products, Ministry of Agriculture of the People's Republic of China, Wuhan, China; ^5^International Research Center for Animal Disease, Ministry of Science and Technology of the People's Republic of China, Wuhan, China

**Keywords:** seneca valley virus, stress granule, 3C protease, eIF4GI-G3BP1 interaction, PKR

## Abstract

Stress granules (SGs) are the sites of mRNA storage and related to the regulation of mRNA translation, which are dynamic structures in response to various environmental stresses and viral infections. Seneca Valley virus (SVV), an oncolytic RNA virus belonging to Picornaviridae family, can cause vesicular disease (VD) indistinguished from foot-and-mouth disease (FMD) and other pig VDs. In this study, we found that SVV induced SG formation in the early stage of infection in a PKR-eIF2α dependent manner, as demonstrated by the recruitment of marker proteins of G3BP1 and eIF4GI. Surprisingly, we found that downregulating SG marker proteins TIA1 or G3BP1, or expressing an eIF2α non-phosphorylatable mutant inhibited SG formation, but this inhibition of transient SG formation had no significant effect on SVV propagation. Depletion of G3BP1 significantly attenuated the activation of NF-κB signaling pathway. In addition, we found that SVV inhibited SG formation at the late stage of infection and 3C protease was essential for the inhibition depending on its enzyme activity. Furthermore, we also found that 3C protease blocked the SG formation by disrupting eIF4GI-G3BP1 interaction. Overall, our results demonstrate that SVV induces transient SG formation in an eIF2α phosphorylation and PKR-dependent manner, and that 3C protease inhibits SG formation by interfering eIF4GI-G3BP1 interaction.

## Introduction

Seneca Valley virus (SVV) is a non-enveloped positive single-stranded RNA virus that belongs to the family *Picornaviridae* (1, 2). SVV was detected as a cell culture contaminant in 2002 in the United States and subsequently identified as a novel picornavirus closely related to members of the genus Cardiovirus (1). SVV genome contains a single open reading frame (ORF) consisting of 6543 nt encoding a polyprotein, which is cleaved into 4 structural proteins forming the viral capsid and 8 non-structural proteins playing indispensable roles on viral replication (1). Since 2007, SVV infection has been a serious threat to the global swine industry and there is no effective vaccine to control the disease (3, 4). Regardless of its harm to swine industry, SVV has been tested as an oncolytic agent for human cancer treatment, which has been tested in phase II clinical trials. A better understanding of virus-host interactions will contribute to disease prevention and control and the improvement of the virus-based therapeutics for cancer treatment.

Virus infection results in various cellular stress responses that modulate cellular gene expression by influencing the mRNA translation, localization and degradation (5). SG formation is one of the stress responses. There are four stress kinases involved in SG formation: double-stranded RNA (dsRNA)-dependent protein kinase (PKR) is activated by dsRNA during viral infection; protein kinase R-like endoplasmic reticulum kinase (PERK) senses unfolded proteins accumulation in the endoplasmic reticulum; heme-regulated inhibitor kinase (HRI) is activated when heme levels are changed by arsenite; and general control non-derepressible 2 kinase (GCN2) is activated under amino acid starvation (6). The activation of these kinases induces the phosphorylation of the alpha-subunit of eukaryotic initiation factor 2 (eIF2α), which shuts up most of initiation of eukaryotic translation and inhibits the formation of eIF2-GTP-${\text{tRNA}}_{\text{i}}^{\text{Met}}$ ternary complex (7). The initiation of mRNA translation is suppressed and polysomes are disassembled during SGs formation. Canonical SGs consist of translationally silent mRNA, 40S ribosomal subunits, eukaryotic initiation factors (eIFs) and multiple RNA-binding proteins (RBPs) such as Ras-GTPase-activating protein SH3-domain-binding protein 1 (G3BP1) and T-cell internal antigen 1 (TIA1), and G3BP1 and TIA1 play a pivotal role in SG formation (8, 9). However, SG formation can also be induced in an eIF2α-independent manner by the natural product pateamine A, which inhibits ribosome recruitment and hydrogen peroxide-induced oxidative stress (10–12).

Several studies have reported the relationship between virus infection and SG formation. During virus infection, SG formation is classified into 4 different patterns: no SG formation, stable SG formation, transient SG formation and alternate SG formation (13). Several viruses, such as Porcine reproductive and respiratory syndrome (PRRSV), Rabies virus (RV), and Newcastle disease virus (NDV), induce stable SG formation (14–16). Due to the inhibitory effect of SGs on majority of virus infection, many viruses have evolved various strategies to inhibit SG formation to promote efficient viral propagation. Influenza A virus (IAV) NS1 protein binds to dsRNA to inhibit the activation of PKR, resulting in the blockage of SG formation (17). Japanese Encephalitis Virus (JEV) Core protein interacts with Caprin-1 to block SG formation, thus promoting virus propagation (18). Feline calicivirus (FCV) blocks SG formation through the cleavage of G3BP1 by NS6 protein (19). Picornavirus 2A or L protein inhibits tSG formation by interfering with the eIF4GI-G3BP interaction (20). In addition, ECMV infection induces transient SG formation on account of the cleavage G3BP1by L protein (13).

In the present study, we show that SVV infection induces transient SG formation in a PKR-eIF2α dependent manner. Although transient SG formation has no effect on SVV replication, SG core component G3BP1 is critical for the expression of cytokine genes induced by SVV. Importantly, our data indicate that SVV 3C blocks the SG formation by disrupting eIF4GI-G3BP1 interaction.

## Materials and Methods

### Cells and Viruses

HEK 293T cells were cultured in Dulbecco's modified essential medium (DMEM; Hyclone, USA) containing 10% fetal bovine serum (Gibco, USA) and 100 U/ml of penicillin (Sigma, USA) at 37°C in a humidified 5% CO2 incubator. SK6 cells were grown in MEM containing 10% FBS, 100 U/ml of penicillin grown under the same conditions as those described above. The SVV strain HB-CH-2016 (GenBank accession number KX377924) used in this study was isolated from piglets with PIVD (21). Virus was propagated and virus titers were determined using a PFU assay in BHK21 cells.

### Plasmid and Reagent

The full-length coding sequences (CDS) of individual SVV proteins were obtained from SVV (KX377924) cDNA. VP1, VP2, VP3, 2C, 3C, and 3D protein were cloned into vector pCAGGS-HA. Lpro, 2B, and 3A protein were cloned into vector pEBG-GST. The coding sequences (CDs) of G3BP1, TIA1 and eIF2α were amplified from 293T cells cDNA and then cloned into vector pCAGGS-HA. Mutant HA-eIF2α-S51A, HA-3C-H48A, HA-3C-C160A, and HA-3C-DM were generated using overlap PCR.

For positive SG formation, 293T cells were treated with 0.5 mM SA (Sigma-Aldrich) for 50 min or incubated at 45°C for 1 h or transfected with poly I:C for 12 h, and then further analysis was conducted. 50 μg/ml CHX (MCE) was used to inhibit canonical SG formation.

### Western Blot and Co-IP Analysis

Cells were harvested and treated with lysis buffer (1.19% HEPES, 0.88% NaCl, 0.04% EDTA, 1% NP-40) containing a protease inhibitor (Roche, UK), and then incubated on ice for 30 min. Equal amounts of protein was subjected to 12% sodium dodecyl sulfate polyacrylamide gel electrophoresis (SDS-PAGE) and then the separated protein was transferred onto polyvinylidene fluoride (PVDF) membranes (Roche, UK). In the co-immunoprecipitation assay, the cell lysates were immunoprecipitated with the indicated antibodies at 4°C for night. And then antibodies-protein complex was incubated with beads at 4°C for 6 h. Subsequently, the complex was washed five times with cold lysis buffer, and then an immunoblotting analysis was conducted.

Mouse monoclonal or rabbit polyclonal antibodies against Flag tag (M385-3L) and HA tag (M180-3) were purchased from Medical and Biological Laboratories (MBL). Alexa Fluor 555 goat anti-mouse or -rabbit antibodies and Alexa Fluor 488 goat anti-rabbit or -mouse antibodies were obtained from Invitrogen. Mouse anti-GAPDH (60004-1-Ig), anti-β-actin (60008-1-Ig) and anti-α-tubulin (66031-1-Ig) monoclonal antibody were obtained from ProteinTech Group. Rabbit polyclonal antibodies against eIF4GI (A0881), PKR (A5578), phosphor-PKR (AP1134), eIF2α (A0764), and phosphor-eIF2α (AP0692) were obtained from ABclonal. Rabbit polyclonal antibodies against G3BP1 (GTX112191) were purchased from Genetex.

### Lentivirus Packaging

The shRNA constructs were designed by using the pLKO.1 vector in accordance with the manufacturer's instructions. HEK293T cells were cotransfected with 0.2 μg pCMV-VSVg, 0.8 μg pCMV-Gag/pol, and 1 μg PLKO.1 for 48 h to generate lentivirus (22). The medium was harvested and centrifuged at 10,000 rpm for 5 min and then divided and stored at −80 °C. When the lentivirus was used for infection, 293T cells were seeded in 12-well plates for 24 h, and then lentivirus containing 1 μg/ml polybrene was added. After 16 h incubation, the medium was replaced with fresh complete growth media for another 48 h. Primer pairs used in this study are listed in Supplementary Table 1.

### RT-qPCR

Total RNA was extracted from the targeted cells using TRIzol reagent (Invitrogen) in accordance with the manufacturer's instructions. One Microgram total RNA was reverse transcribed to cDNA using HiScript III 1st Strand cDNA Synthesis Kit (Vazyme). The relative mRNA level of targeted gene was measured with qPCR using SYBR green real-time PCR master mix (Applied Biological Materials Inc) with specific primer (Supplementary Table 1). All reactions were performed in triplicate. The mRNA level of housekeeping gene GAPDH was used as an internal control. Relative gene fold was determined with the comparative cycle threshold (2^−ΔΔCT^) method.

### Immunofluorescence Analysis

Cells were fixed with 4% paraformaldehyde for 30 min and permeated with 0.2% Triton X-100 at room temperature for 20 min, and then blocked with 3% bovine serum albumin (BSA) in phosphate-buffered saline (PBS) for 1 h. Subsequently, the primary antibodies were diluted in PBS at indicated concentration at 37°C for 2 h. After being washed with PBS for five times, the cells were incubated with secondary antibodies at room temperature for 1 h. The cells were washed with PBS for five times and incubated with 4'6-diamidino-2-phenyl-indole (DAPI; Beyotime Biotechnology) for 10 min. Fluorescent images were acquired with a confocal laser scanning microscope (LSM 510 Meta; Carl Zeiss).

### Statistical Analysis

Statistical analysis was conducted using GraphPad Prism software, version 5. All results are determined by at least three times independent experiments. The various treatments were compared using an unpaired, two-tailed Student *t*-test with an assumption of unequal variance. *P* < 0.05 was considered statistically significant.

## Result

### SVV Infection Induces Transient SG Formation Relying on Viral Replication

**Figure 1:**
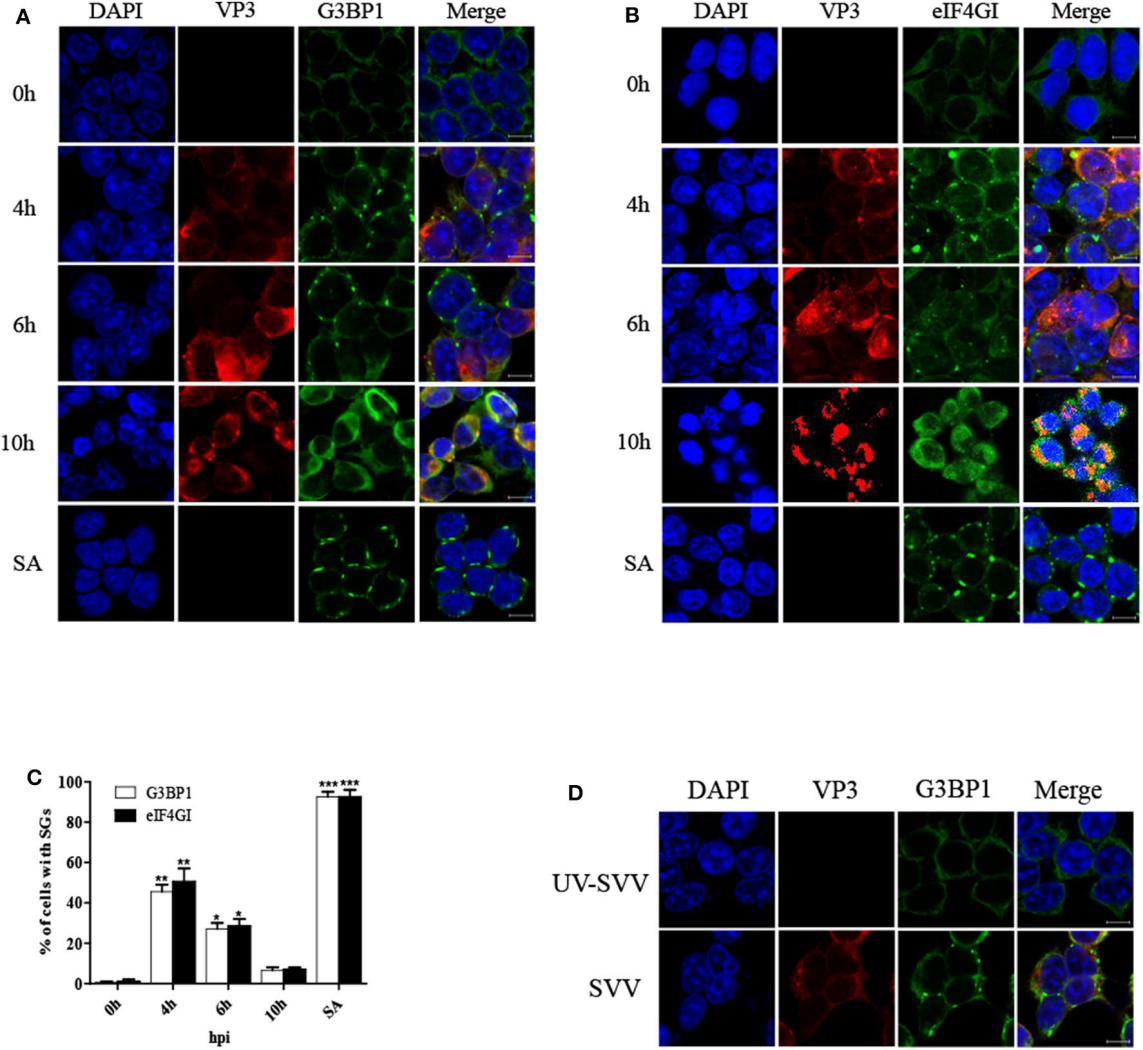
SVV infection induces transient SG formation relying on viral replication. **(A,B)** 293T cells were mock-infected or infected with SVV (MOI = 1) at the indicated time points. And cells were treated with SA (0.5 mM) for 50 min as a positive control. The cells were fixed and stained with either rabbit polyclonal specific antibodies for G3BP1 (green) and mouse monoclonal specific antibodies for VP3 (red) or either rabbit polyclonal specific antibodies for eIF4GI (green) and mouse monoclonal specific antibodies for VP3 (red). Nuclei were stained with DAPI (blue). Scale bar = 10 μm. Then cells were analyzed by confocal microscopy. **(C)** The percentage of cells containing SGs was calculated in three independent experiments. At least 100 cells were counted each time. Data are represented as means ± SD. Student's *t*-test: *P < 0.05, **P < 0.01, ***P < 0.001, ns = not significant. **(D)** 293T cells were infected with SVV or UV-inactivated SVV (MOI = 1) for 4 h. The cells were analyzed by confocal microscopy after staining with anti-VP3 antibodies and anti-G3BP1 antibodies.

To determine the formation of SG during SVV infection, 293T cells were infected with SVV at different time post infection and the distribution of SG marker protein G3BP1 was examined through performing indirect immunofluorescence assays. The cells treated with sodium arsenite (SA) were considered as a positive control. The antibody against SVV VP3 was used to detect viral replication. Immunofluorescence analysis revealed that SVV infection induced G3BP1 cytoplasmic foci at the early stage of infection, and then the gradual disaggregation of G3BP1 cytoplasmic foci was observed as time progressed (Figure 1A). To further confirm the result, we also investigated the distribution of eIF4GI (another SG marker) in SVV-infected cells. The distribution of eIF4GI was found to be consistent with that of G3BP1 (Figure 1B). The dynamics of SG formation revealed that the percentage of the cells containing SGs increased to ~50% at 4 hpi, then decreased gradually and few SGs were observed at 10 hpi (Figure 1C). To examine whether SVV infection was indispensable for the transient SG formation, UV-inactivated SVV was performed. Loss of infectivity was determined by detecting viral VP3 protein. 293T cells were infected with SVV or UV-inactivated SVV for 4 h. Then the cells were fixed and stained with antibodies against G3BP1 and SVV VP3. As showed in Figure 1D, unlike SVV infection, UV-inactivated SVV infection triggered no SG formation. These results indicate that SVV infection induces transient SG formation relying on viral replication.

### CHX Treatment Disrupts SVV-Induced SG Formation

**Figure 2:**
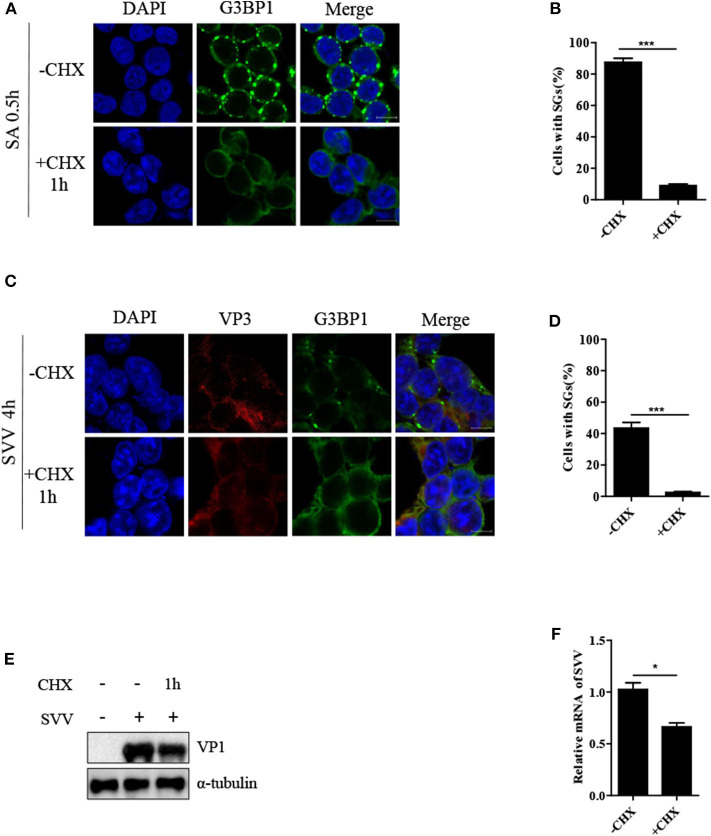
CHX treatment disrupts SVV-induced SGs. **(A)** 293T cells were treated with 0.5 mM SA for 0.5 h and then treated with or without CHX for another 1 h. The cells were fixed and subsequently immunostained for G3BP1 (green). Nuclei were stained with DAPI (blue). Scale bar = 10 μm. **(B)** The percentage of cells containing SGs was calculated in three independent experiments. At least 100 cells were counted each time. **(C)** 293T cells were infected with SVV for 4 h and then treated or untreated with CHX for another 1 h before indirect immunofluorescence was performed. Scale bar = 10 μm. **(D)** The percentage of cells containing SGs were calculated (at least 100 cells). **(E)** 293T cells were infected with SVV for 4 h and then treated or untreated with CHX for another 1 h. The cell lysates were analyzed by western blot using anti-VP1 and anti-α-tubulin antibodies. **(F)** Totol RNA was extracted and the relative amount of mRNA of SVV 5'-UTR was determined by RT-qPCR and β-actin was considered as housekeeping genes. Data are represented as means ±SD. Student's *t*-test: *P < 0.05, **P < 0.01, ***P < 0.001, ns = not significant.

Cycloheximide (CHX), a global inhibitor of protein synthesis, traps mRNAs in polysomes by blocking translational elongation and inhibits SG formation (23). SA-induced SGs were disassembled in the presence of CHX for 1 h (Figures 2A,B). Some virus-triggered SGs exhibited specific feature, compared with canonical SGs. Unlike canonical SGs, RV-induced SGs were not disassemble when the cells were incubated with CHX for 1 h (15). To determine whether SVV-induced transient SGs were disassembled in response to CHX treatment, the cells were infected with SVV for 4 h and then incubated with CHX for an additional 1 h. As expected, SVV-induced SGs dramatically decreased in the presence of CHX (Figures 2C,D). Since CHX is a translational inhibitor of protein synthesis, we investigated the expression level of viral VP1 in the presence or absence of CHX. As shown in Figure 2E, VP1 protein expression was inhibited in the presence of CHX. To detect whether viral mRNA synthesis rate was affected through CHX treatment, we examined the level of viral mRNA incubated with CHX or not, and found that viral mRNA level obviously decreased in the presence of CHX (Figure 2F). Taken together, SVV-induced SGs are similar to canonical SGs.

### SVV Triggers SG Formation via PKR-eIF2α Signaling Pathway

**Figure 3:**
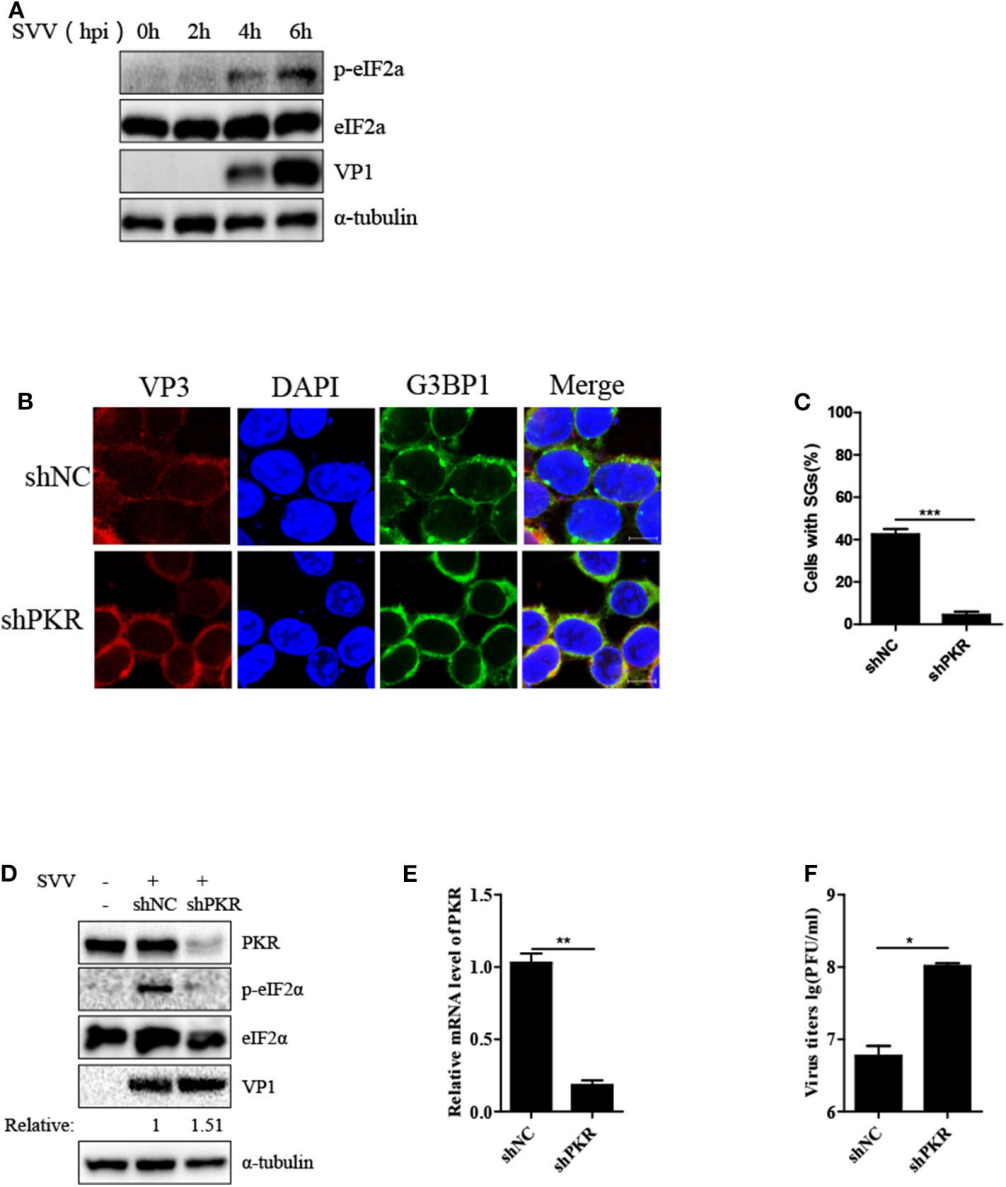
SVV triggers SG formation via PKR-eIF2α signaling pathway. **(A)** 293T cells were mock-infected or infected with SVV at a MOI of 1 at the indicated time point. The cell lysates were analyzed by western blot using anti-VP1, anti-eIF2α, anti-phosphorylated eIF2α, and anti-α-tubulin antibodies. **(B)** 293T cells were transducted with shPKR lentivirus and shNC lentivirus for 48 h and then infected with SVV at 1 MOI for 4 h. The cells were fixed and stained with either rabbit polyclonal specific antibodies for G3BP1 (green) and mouse monoclonal specific antibodies for VP3 (red). Nuclei were stained with DAPI (blue). Scale bar = 10 μm. **(C)** The percentage of cells containing SGs was calculated in three independent experiments. At least 100 cells were counted each time. **(D)** 293T cells were transducted with shPKR lentivirus and shNC lentivirus for 48 h and then infected with SVV at 1 MOI for 8 h. The cell lysates were analyzed by western blot using anti-VP1, anti-PKR, anti-eIF2α, anti-phosphorylated eIF2α, and anti-α-tubulin antibodies. **(E)** Totol RNA was extracted and the relative amount of mRNA of PKR was determined by RT-qPCR and GADPH was considered as housekeeping genes. **(F)** The supernatants were harvested and the virus titers were determined by plaque assay. Data are represented as means ±SD. Student's *t*-test: *P < 0.05, **P < 0.01, ***P < 0.001, ns = not significant.

We further examined the manner in which SVV induced SG formation. As the phosphorylation of eIF2α was pivotal for SG formation, we assessed the phosphorylation of eIF2α level at different time points after SVV infection. As shown in Figure 3A, the level of phosphorylation of eIF2α increased at 4 hpi and increased in a time-dependent manner. SVV was reported to be a positive single-stranded RNA virus that produces significantly abundant dsRNA during replication (1). Therefore, we further determined whether PKR was indispensable for SVV-induced SG formation, which can be activated by dsRNA and then resulted in the phosphorylation of eIF2α. Lentivirus was used to downregulate the expression of PKR. The depletion effect of PKR was demonstrated by the results of RT-qPCR (Figure 3E). In shNC-293T cells, SVV infection induced SG formation. In contrast, SVV-triggered SGs were blocked in shPKR-293T cells (Figures 3B,C).

Subsequently, we investigated the phosphorylation level of eIF2α in shNC-293T and shPKR-293T cells after SVV infection and found that depletion of PKR blocked the phosphorylation of eIF2α, indicating that SVV-triggered eIF2α phosphorylation depended on PKR (Figure 3D). Interestingly, silencing PKR expression increased viral VP1 protein expression and virus titer (Figures 3D,F). PKR is pivotal for type I interferon production and host antiviral response (24). And SVV is an IFN-β-sensitive virus (25). This may be the reason for antiviral effect of PKR to SVV. Taken together, these results suggest that PKR is critical for SVV-induced SG formation and plays an inhibitory role in SVV replication.

### Inhibition of SG Formation Has no Significant Effect on SVV Replication

**Figure 4:**
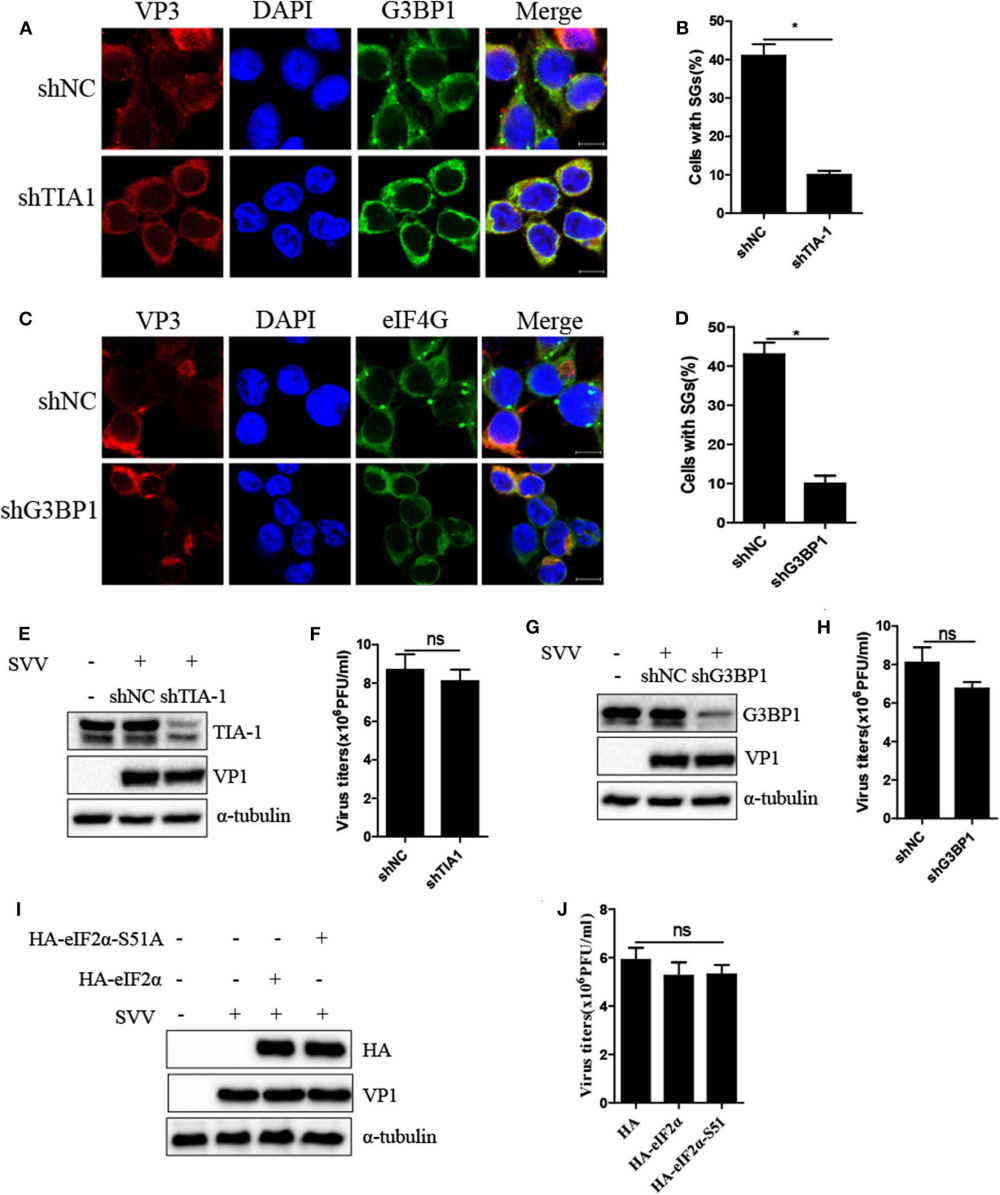
Inhibition of SG formation has no significant effect on SVV replication. **(A)** 293T cells were transducted with shTIA1 lentivirus and shNC lentivirus for 48 h and then infected with SVV at 1 MOI for 4 h. The cells were fixed and stained with either rabbit polyclonal specific antibodies for G3BP1 (green) and mouse monoclonal specific antibodies for VP3 (red). Nuclei were stained with DAPI (blue). Scale bar = 10 μm. **(B)** The percentage of cells containing SGs was calculated in three independent experiments. At least 100 cells were counted each time. **(C)** 293T cells were transducted with shG3BP1 lentivirus and shNC lentivirus for 48 h and then infected with SVV at 1 MOI for 4 h. The cells were fixed and stained with either rabbit polyclonal specific antibodies for eIF4GI (green) and mouse monoclonal specific antibodies for VP3 (red). Nuclei were stained with DAPI (blue). Scale bar = 10 μm. **(D)** The percentage of cells containing SGs was calculated in three independent experiments. **(E,F)** 293T cells were transducted with shTIA1 lentivirus and shNC lentivirus for 48 h and then infected with SVV at 1 MOI for 6 h. The cell lysates were analyzed by western blot using anti-TIA1, anti-VP1, and anti-α-tubulin antibodies. The supernatants were harvested and the virus titers were determined by plaque assay. **(G,H)** 293T cells were transducted with shG3BP1 lentivirus and shNC lentivirus for 48 h and then infected with SVV at 1 MOI for 6 h. The cell lysates were analyzed by western blot using anti-G3BP1, anti-VP1, and anti-α-tubulin antibodies. The supernatants were harvested and the virus titers were determined by plaque assay. **(I)** 293T cells were transfected with vector, HA-eIF2α, or HA-eIF2α-S51A for 20 h and then infected with SVV at 1 MOI for another 6 h. The cell lysates were analyzed by western blot using anti-VP1, anti-TIA1, and anti-α-tubulin antibodies. **(J)** The supernatants were harvested and the virus titers were determined by plaque assay. Data are represented as means ±SD. Student's *t*-test: *P < 0.05, **P < 0.01, ***P < 0.001, ns = not significant.

Virus-triggered SGs or SG components play pivotal roles in virus replication (26). To determine whether SG formation exhibited certain effect on viral propagation, we abolished transient SG formation through depletion of pivotal SG component TIA1 or G3BP1. As shown in Figures 4A–D, the percentage of SGs in TIA1-knockdown or G3BP1-knockdown cells substantially decreased after SVV infection, indicating that depletion of TIA1 or G3BP1 significantly disrupted SG formation. Next, we investigated the level of SVV VP1 protein expression and virus titers. Surprisingly, we found that blocking SG formation exhibited no significant influence on SVV replication (Figures 4E–H). On account of SVV-induced SG formation via PKR-eIF2α signaling pathway, we overexpressed HA-eIF2α-S51A (HA-tagged non-phosphorylatable mutant of eIF2α) to inhibit SVV-triggered SGs. Similarly, overexpressing HA-eIF2α or HA-eIF2α-S51A had no effect on the expression viral VP1 (Figure 4I) and virus production (Figure 4J). Taken together, these results indicate that SVV-induced transient SG formation has no significant influence on SVV replication.

### The Key Component of SG G3BP1 Is Critical for SVV-Induced Activation of NF-κB Signaling Pathway

**Figure 5:**
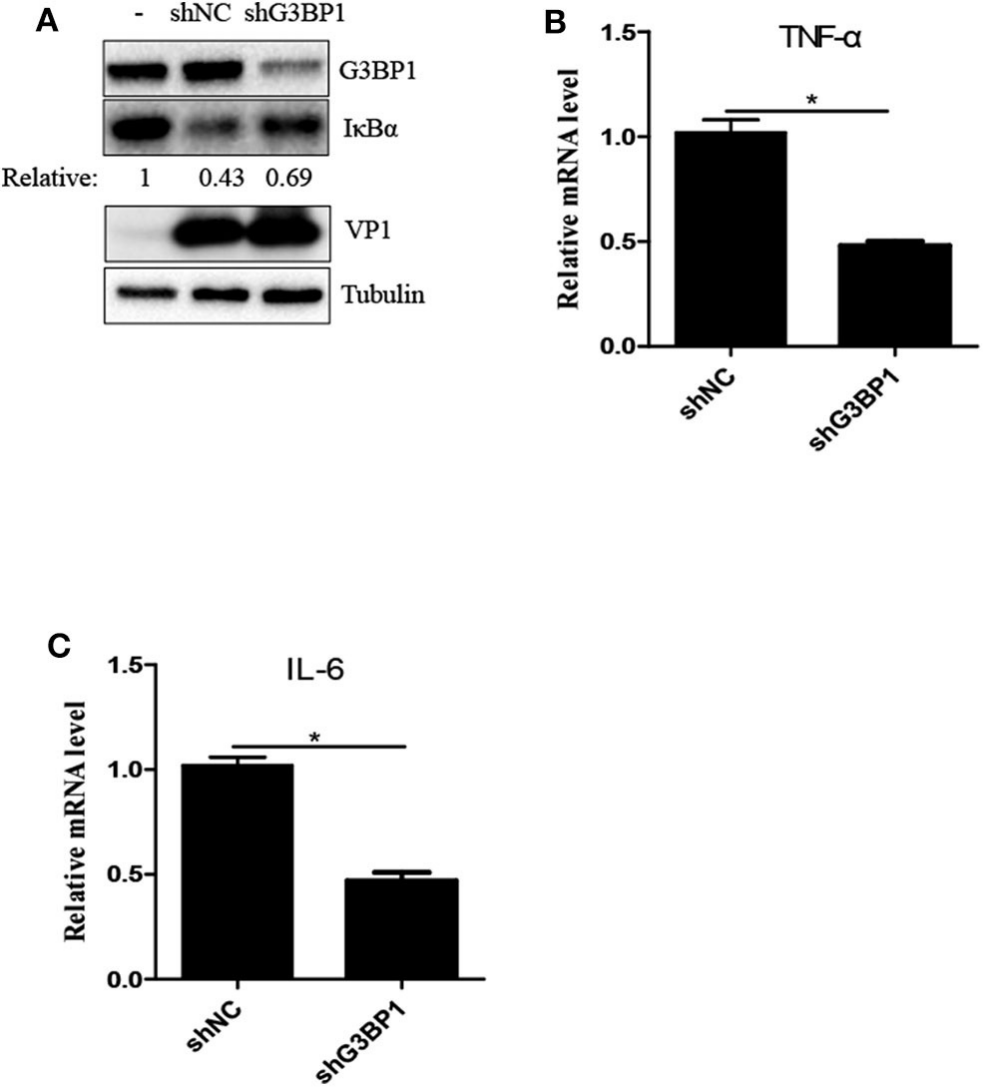
The SG key component G3BP1 is critical for SVV-induced activation of NF-κB signaling pathway. **(A)** 293T cells were transducted with shG3BP1 lentivirus and shNC lentivirus for 48 h and then infected with SVV at 1 MOI for 8 h. The cell lysates were analyzed by western blot using anti-VP1, anti-G3BP1, anti-IκBα and anti-α-tubulin antibodies. **(B,C)** Totol RNA was extracted and the relative amount of mRNA of IL-6 and TNFα was determined by RT-qPCR and β-actin was considered as housekeeping genes. Data are represented as means ±SD. Student's *t*-test: *P < 0.05, **P < 0.01, ***P < 0.001, ns = not significant.

Previous study reported that SVV infection activated NF-κB signaling pathway (25, 27). G3BP1-induced SGs are linked to the activation of innate immune transcriptional responses through NF-κB and JNK (28). To investigate whether G3BP1 had a biological impact on activation of NF-κB in SVV-infected cells, 293T cells were transducted with shG3BP1 lentivirus for 48 h and then infected with SVV for another 8 h. Interestingly, the protein level of IκBα was degraded in the presence of SVV and the degradation degree was obviously rescued when G3BP1 expression was downregulated (Figure 5A). At the same time, the mRNA level of pro-inflammasome cytokines was detected by RT-qPCR. The mRNA level of IL-6 and TNFα in SVV-infected cells depleted G3BP1 were significantly decreased, compared to those in SVV-infected cells transducted with shNC lentivirus (Figures 5B,C). Taken together, our data suggest that SG component G3BP1 is important for SVV-induced activation of NF-κB signaling pathway.

### SVV 3C Inhibits SA-Induced SG Formation Depending on Its Protease Activity

**Figure 6:**
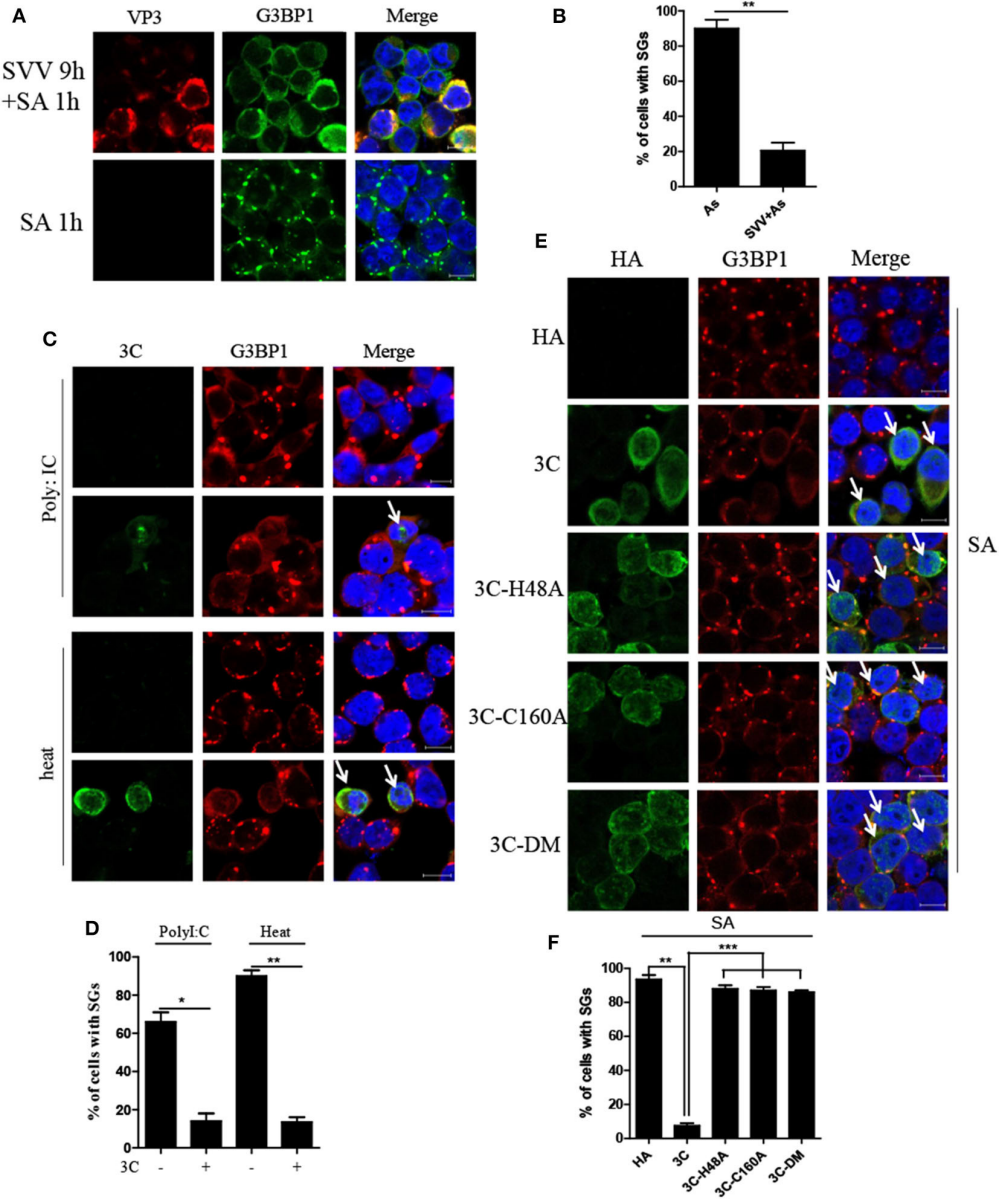
SVV 3C inhibits SA-induced SG formation depending on its protease activity. **(A)** 293T cells were infected or uninfected with SVV at 1 MOI for 9 h and subsequently treated with 0.5 mM SA for another 50 min. The cells were fixed and stained with either rabbit polyclonal specific antibodies for G3BP1 (green) and mouse monoclonal specific antibodies for VP3 (red). Nuclei were stained with DAPI (blue). Scale bar = 10 μm. **(B)** The percentage of cells containing SGs was calculated in three independent experiments. At least 100 cells were counted each time. **(C)** 293T cells were transiently transfected with vector or HA-3C for 20 h and then stimulated with heat shock at 45°C for 50 min or poly I:C for 12 h. The cells were fixed and stained with either rabbit polyclonal specific antibodies for G3BP1 (red) and mouse monoclonal specific antibodies for HA (green). Nuclei were stained with DAPI (blue). Scale bar = 10 μm. **(D)** The percentage of 3C-positive cells containing SGs was calculated in three independent experiments. At least 100 cells were counted each time. **(E)** 293T cells were transiently transfected with vector, HA-3C, or HA-3C variants for 20 h and then treated with 0.5 mM SA for another 50 min. **(F)** The percentage of 3C- or 3C mutants-positive cells containing SGs was calculated in three independent experiments. At least 100 cells were counted each time. Data are represented as means ± SD. Student's *t*-test: *P < 0.05, **P < 0.01, ***P < 0.001, ns = not significant.

Considering that SVV induced transient SG formation, we speculated that SVV contained certain mechanism to inhibit SG formation during infection. To confirm whether SVV infection could block SG formation in late infection in response to sodium arsenite (SA), we examined SA-induced SG formation in mock- or SVV-infected cells. In mock-infected cells, SA-induced SGs were detected in about 90% of mock-infected cells, whereas, they were detected in only ~20% of SVV-infected cells (Figures 6A,B), indicating that SVV infection could block SA-induced SG formation in late infection. To reveal how SVV blocked SG formation, 293T cells were transfected with viral proteins or an empty vector for 24 h and then stimulated with 0.5 mM SA for 50 min. We found that SVV 3C could block SA-induced SG formation (Supplementary Figure 1). In addition, we conducted similar experiments under the stress condition of heat shock or poly I:C stimulation and found that SVV 3C also showed a strong ability to inhibit heat- or poly I:C-induced SG formation (Figures 6C,D). Previous studies showed that single-site mutant H48A (3C-H48A) or C160A (3C-C160A) and double site mutant H48A-C160A (3C-DM) were catalytically inactive (25). Interestingly, we found that SVV 3C mutants with abolition of protease activity lost the ability to block SA-induced SG formation, indicating that 3C protease activity was indispensable for its disruption of SA-induced SG formation (Figures 6E,F). Collectively, SVV 3C inhibits SA-induced SG formation depending on its protease activity.

### SVV 3C Inhibits SG Formation by Disrupting G3BP1-eIF4GI Interaction

**Figure 7:**
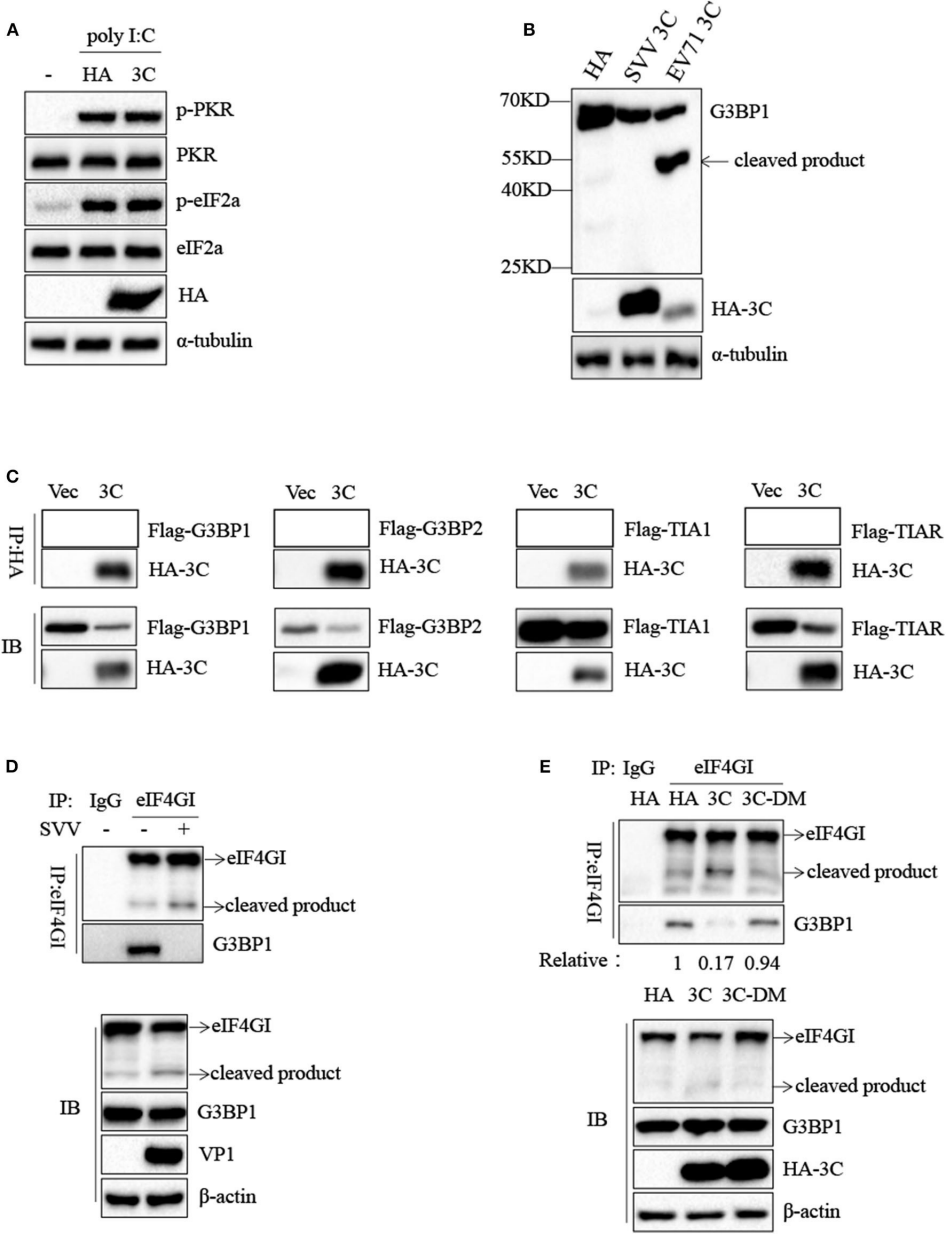
SVV 3C inhibits SG formation by disrupting G3BP1-eIF4GI interaction. **(A)** 293T cells were transfected with vector or HA-3C for 12 h and subsequently transfected with poly I:C for another 12 h. The cell lysates were analyzed by western blot using anti-VP1, anti-phosphorylated PKR, anti-PKR, anti-eIF2α, anti-phosphorylated eIF2α, and anti-α-tubulin antibodies. **(B)** 293T cells were cotransfected with SVV 3C or EV71 3C and G3BP1 for 24 h. The cell lysates were analyzed by western blot using anti-Flag, anti-HA, and anti-α-tubulin antibodies. **(C)** 293T cells were cotransfected with G3BP1, G3BP2, TIA1, or TIAR and SVV 3C for 24 h. Subsequently, the interaction between G3BP1, G3BP2, TIA1, or TIAR and SVV 3C was measured by anti-Flag immunoprecipitation. **(D)** 293T cells were infected or uninfected with SVV at 1 MOI for 8 h, and the cell lysates were subjected to immunoprecipitation with anti-eIF4GI antibodies. **(E)** 293T cells were transfected with vector, 3C, or 3C-DM and eIF4GI for 24 h and then infected or uninfected with SVV at 1 MOI for 8 h. And the cell lysates were subjected to immunoprecipitation with anti-eIF4GI antibodies.

To confirm the underlying mechanism by which SVV 3C inhibited SG formation, we firstly investigated whether SVV 3C inhibited the phosphorylation of PKR and eIF2α. As shown in Figure 7A, SVV 3C had no effect on the phosphorylation of PKR and eIF2α stimulated with poly I:C. Previous studies showed that SG inhibition can be produced by the interaction, sequestration, or cleavage of the SG component. Picornavirus 3C protease targeted G3BP1 for cleavage to disrupt the assembly of SG (13, 29). SVV 3C cleaved cellular proteins such as MAVS, TRIF and TANK to regulate virus propagation (25). Therefore, we investigated whether SVV 3C could induce the cleavage of G3BP1. 293T cells were transfected with vector or SVV 3C and G3BP1 for 24 h and then analyzed by immunoblotting. As shown in Figure 7B, transfection with EV71 3C resulted in the cleavage of G3BP1. However, G3BP1 remained intact in the presence of SVV 3C. In addition, we found that SVV 3C did not interact with G3BP1, G3BP2, TIA1, or TIAR (Figure 7C).

**Figure 8:**
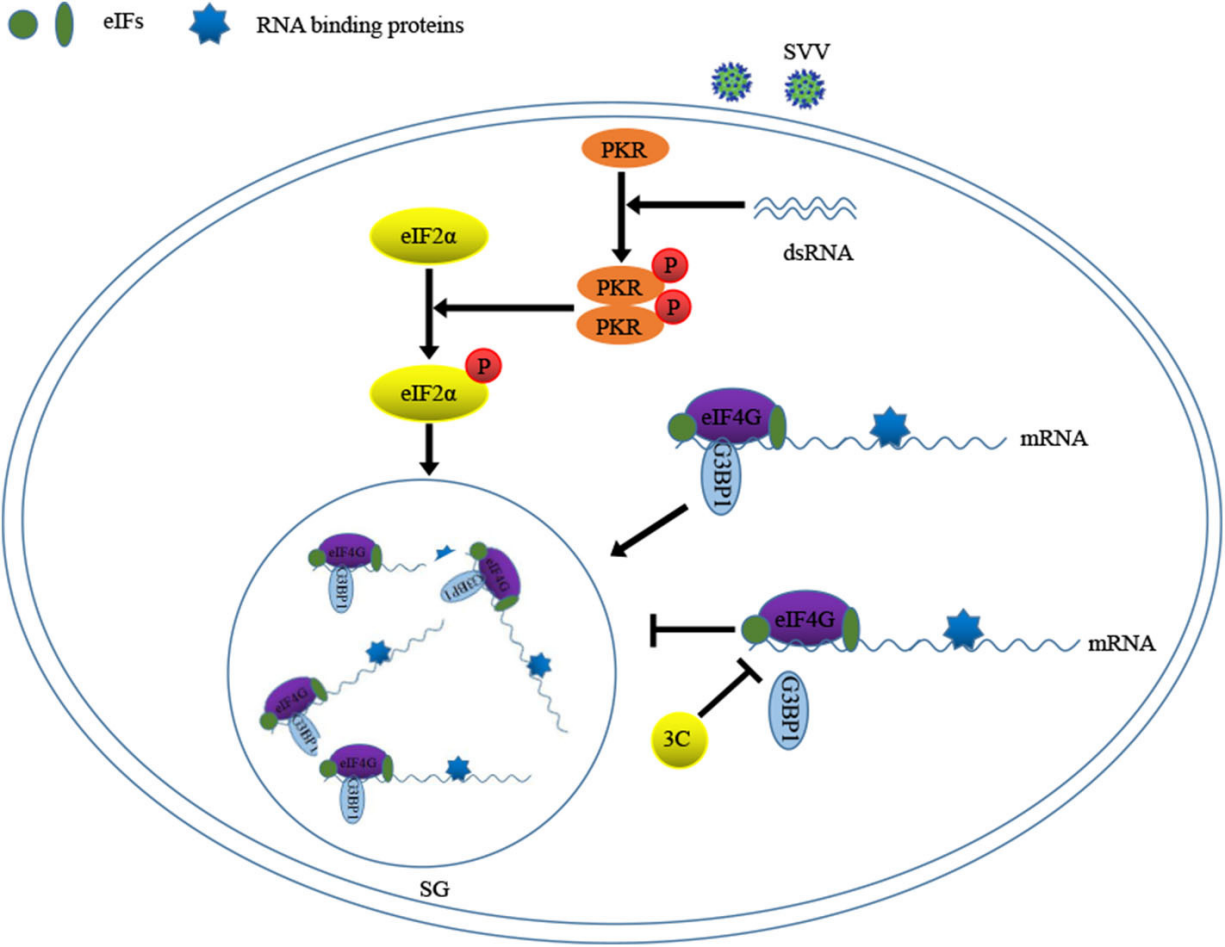
Model of SVV 3C regulation SG formation by G3BP1-eIF4GI interaction.

Previous study showed that 2A or L protein of picornavirus blocked SG formation by disrupting the interaction between eIF4GI and G3BP1 (20). We speculated that SVV 3C might possess similar function to inhibit SG formation. 293T cells were infected with 1 MOI of SVV for 8 h, and then Co-IP assay was performed by using anti-eIF4GI antibody. The results indicated that eIF4GI could interact with G3BP1 in mock cells, whereas eIF4GI failed to interact with G3BP1 in the presence of SVV (Figure 7D). To determine whether SVV 3C played a vital interference in the interaction, 293T cells were transfected with vector or SVV 3C for 24 h and then Co-IP assay was performed. As we expected, SVV 3C disrupted eIF4GI-G3BP1 interaction (Figure 7E). Furthermore, 3C-DM failed to inhibit SG formation and exhibited no obvious inhibitory effect on eIF4GI-G3BP1 interaction (Figure 7E). Though 3C targeted eIF4GI for cleavage relying on its protease activity (Supplementary Figure 2), the cleavage ability was extremely low and the interaction between eIF4GI and G3BP1 was almost undetectable in the presence of 3C. Taken together, our results show that SVV 3C inhibits SG formation by disrupting eIF4GI-G3BP1 interaction (Figure 8).

## Discussion

Virus infection causes various stress responses in host cells, resulting in SG formation. The mechanism of SG formation has been elucidated, nevertheless the function of SGs remains controversial. Abundant data show that SG formation and SG core components play distinct roles in virus propagation (antiviral, proviral or no effect) (14, 30–32). Additionally, many viruses antagonize SG formation to weaken its antiviral effects (13, 18, 33). In this study, we showed that SVV infection induced transient SG formation in a PKR- and eIF2α phosphorylation-dependent manner, and that SVV replication was essential for SG formation. Surprisingly, transient SG formation exhibited no significant effect on SVV replication. Furthermore, SVV 3C inhibited SG formation by disrupting eIF4GI-G3BP1 interaction.

In the results, SVV infection induced SG formation in early infection, whereas the SGs disassembled in late infection (Figures 1A,B), suggesting that viral proteins might adopt a certain strategy to counteract SG formation. Previous studies showed that some virus-triggered SGs were different from canonical SGs. Rabies virus induced non-canonical SGs, which could not be disassembled in the presence of CHX (15). In this study, CHX treatment resulted in the disassembly of SVV-triggered SGs (Figures 2A–C), suggesting that SVV-induced transient SGs were canonical SGs. CHX, a translational inhibitor of protein synthesis, also inhibited SVV VP1 synthesis (Figure 2E).

SVV, a non-enveloped positive single-stranded RNA virus, can generate intermediate double-stranded RNA during replication, which may activate PKR to phosphorylate eIF2α resulting in SG assembly. As expected, downregulation of PKR expression significantly suppressed eIF2α phosphorylation and blocked SG assembly (Figures 3B,C). PKR is of considerable importance to the production of IFN-β and modulation of inflammatory immune responses (24, 34). And IFN-β can significantly inhibit SVV propagation (25). We found that knockdown of PKR expression notably increased viral protein expression and virus propagation **(**Figures 3D,F). We further blocked SG formation by knocking down G3BP1 or TIA1 (SG scafford protein) or overexpressing eIF2α-S51A (an eIF2α non-phosphorylatable mutant). To our surprise, blocking SG formation showed no significant effect on SVV replication (Figure 4), which might be attributed to the fact that the number of SVV-induced SGs was small and their existence time was short. Previous studies showed that G3BP1 could slightly activate ISRE promotor and inhibit enterovirus replication (28, 35), which was inconsistent with our results. G3BP1 also had no effect on PRRSV replication because PRRSV N protein abolished G3BP1 antiviral effect via interaction with G3BP1 to induce G3BP1 phosphorylation. The exact reason why G3BP1 has no influence on SVV replication remains to be further explored. In addition, we found that SG core component G3BP1 was involved in SVV-induced activation of NF-κB signaling pathway. In SVV-infected cells, knockdown of G3BP1 decreased the mRNA levels of IL-6 and TNF-α and degradation of IκBα (Figure 5). Our results were consistent with previous findings that G3BP1 promoted multiple innate immune antiviral responses (28).

Some viruses evolved various strategies to block SG formation during infection, which counteracted its antiviral effects (13, 20, 36, 37). And some viruses exploited SG components to facilitate their replication (16, 38). In this study, SVV inhibited SG formation in response to SA at late stage of infection **(**Figures 6A,B). Further results showed that 3C was indispensable for SVV to block SG assembly in response to SA, poly I:C, or heat **(**Figures 6C,D). Our previous studies showed that SVV 3C contained a conserved catalytic box with His and Cys residues which was essential for its functional properties (25, 39). Therefore, in order to detect whether enzyme activity of 3C was essential for inhibition of SG formation, 293T cells were transfected with 3C or its mutants and then stimulated with SA. Our results showed that 3C mutants losing catalytic activity did not inhibit SG formation (Figures 6E,F). Picornavirus 3C or L protein inhibited SG assembly via targeting G3BP1 for cleavage (29, 40, 41). TMEV L protein prevented the activation of PKR via dampening the responsiveness of PKR to dsRNA, thus inhibiting SG formation (36). To our surprise, no cleavage band was detected when 293T cells were co-transfected with 3C and G3BP1 (Figure 7B). In addition, 3C did not impair the phosphorylation of poly I:C-induced PKR and eIF2α (Figure 7A). Previous study showed that eIF4GI-G3BP1 interaction depending on RNA was critical for SG formation, and that picornavirus L or 2A protease can disrupt eIF4GI-G3BP1 interaction relying on its protease activity, resulting in SG formation blockage (20). Interestingly, we found that SVV infection or 3C protease expression disrupted eIF4GI-G3BP1 interaction. 3C-DM did not block SG formation and had no significant effect on eIF4GI-G3BP1 interaction (Figures 7D,E). Though 3C targeted eIF4GI for cleavage relying on its protease activity (Supplementary Figure 2), the cleavage ability was extremely low and the interaction between eIF4GI and G3BP1 was almost undetectable in the presence of 3C (Figure 7E). We predicted that 3C disrupted eIF4GI-G3BP1 interaction, which might be independent from 3C-induced eIF4GI cleavage or 3C-eIF4GI interaction. Since eIF4GI-G3BP1 interaction is RNA-dependent, further studies are needed to detect whether 3C can disrupt eIF4GI-RNA or G3BP1-RNA interaction or both of them.

In conclusion, we present evidence that SG core component G3BP1 is critical for SVV-induced activation of NF-κB signaling pathway. SVV 3C protease disrupts eIF4GI-G3BP1 interaction to inhibit SG formation. This study reveals the mechanism by which SVV manipulates various stress responses, which will be conducive to the understanding of the pathogenesis of disease and the development of effective oncolytic viruses.

## Data Availability Statement

The original contributions presented in the study are included in the article/supplementary material, further inquiries can be directed to the corresponding author/s.

## Author Contributions

WW drafted the main manuscript and performed the data analysis; WW, QZ, MY, and LQ planned and performed experiments; JH, XL, HC, and PQ were responsible for experimental design. All authors reviewed and agreed the publication of this manuscript.

## Conflict of Interest

The authors declare that the research was conducted in the absence of any commercial or financial relationships that could be construed as a potential conflict of interest.
