# Supplementary material for: Seneca Valley Virus 3C Protease Inhibits Stress Granule Formation by Disrupting eIF4GI-G3BP1 Interaction
Source: Front Immunol. 2020 Sep 29;11:577838. doi: 10.3389/fimmu.2020.577838 (PMC7550656; doi:10.3389/fimmu.2020.577838)
Supplement: Supplementary file 1 [file Table_1.DOCX]

Supplementary Material

# Supplementary Figures and Tables

## Supplementary Figures


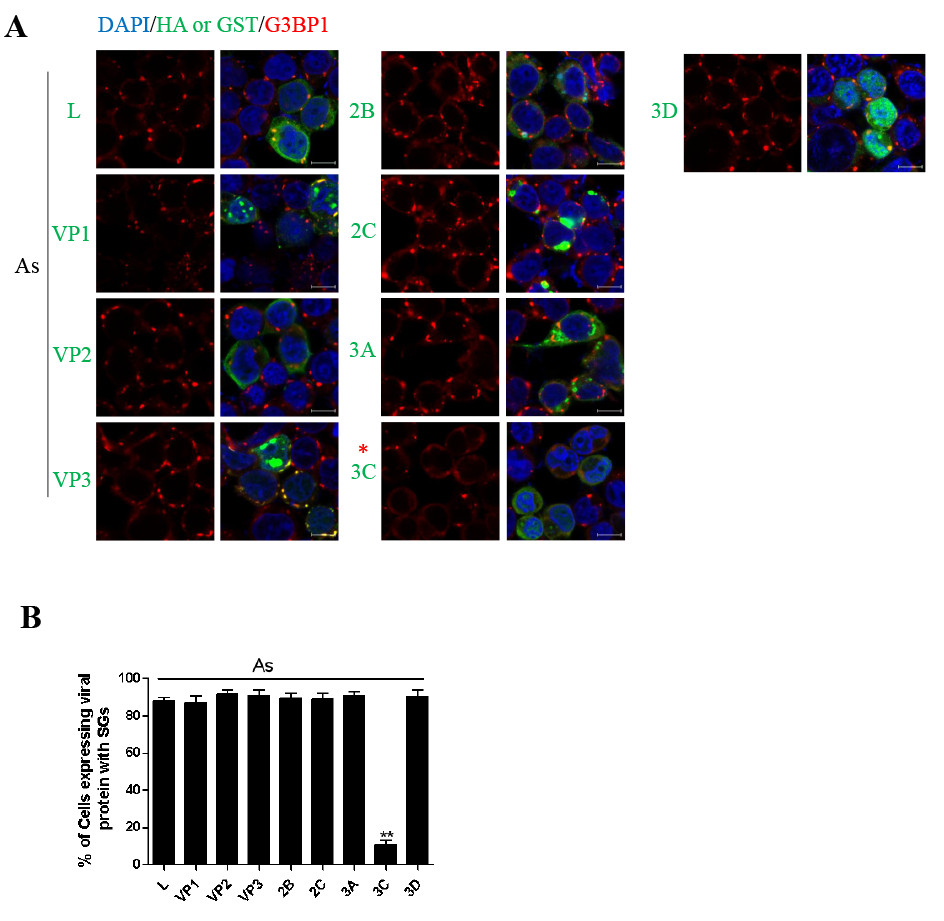


**Supplementary Figure 1. SVV 3C inhibits As-induced SGs formation.** (A and B) 293T cells were transfected with plasmids expressing the indicated SVV proteins for 24 h and then stimulated with SA for 1 h. The cells were fixed and stained with either rabbit polyclonal specific antibodies for G3BP1 (red) and mouse monoclonal specific antibodies for VP3 (green). Nuclei were stained with DAPI (blue). Scale bar = 10 μm. Then cells were analyzed by confocal microscopy


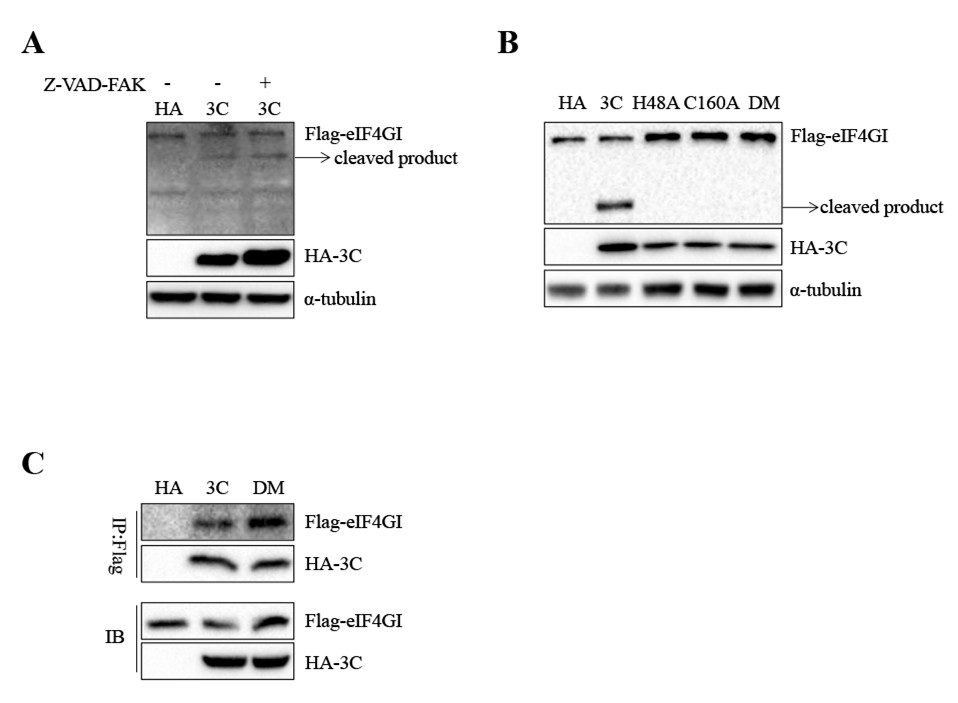


**Supplementary Figure 2. SVV 3C cleaves eIF4GI through its protease activity.** (A) 293T cells were transfected with 3C and eIF4GI for 16 h, and then the cells were treated with 50 μM Z-VAD-FMK or not for another 8 h. The cells were collected for western blot analysis. (B) 293T cells were transfected with 3C or 3C mutants and eIF4GI for 24 h. And then the cells were collected for western blot analysis. (C) 293T cells were transfected with 3C or 3C-DM and eIF4GI for 24 h. The cell lysates were subjected to immunoprecipitation with mouse anti-HA antibody, and the input and IP samples were analyzed by immunoblotting using rabbit anti-Flag antibodies and rabbit anti-HA antibodies.

## Supplementary Tables

**Supplementary Table 1. Primer pairs used in this study**

| Primer | Sequence (5’ to 3’) |
| --- | --- |
| SVV 5’UTR-F | ATGCCCAGTCCTTCCTTTC |
| SVV 5’UTR-R | CAGGCAGTATCCAAAGCACG |
| GDPDH-F | GAGTCAACGGATTTGGTCGT |
| GDPDH-R | GACAAGCTTCCCGTTCTCAG |
| TNFα-F | CCGAGTGACAAGCCTGTAG |
| TNFα-R | GGTCTGGTAGGAGACGGCG |
| IL-6-F | CCAGGAGCCCAGCTATGAAC |
| IL-6-R | CTGAGATGCCGTCGAGGATG |
| PKR-F | GCATGGGCCAGAAGGATTTC |
| PKR-R | GGTAGTCAGATTTCACTGAG |
| shG3BP1 | GCCTGTAAGAAATACAGGATT |
| shPKR | GAGGCGAGAAACTAGACAAAG |
| shTIA1 | GCCGTTGTTTACTTAAAGATT |
